# Eating Dinner Early Improves 24-h Blood Glucose Levels and Boosts Lipid Metabolism after Breakfast the Next Day: A Randomized Cross-Over Trial

**DOI:** 10.3390/nu13072424

**Published:** 2021-07-15

**Authors:** Kaho Nakamura, Eri Tajiri, Yoichi Hatamoto, Takafumi Ando, Seiya Shimoda, Eiichi Yoshimura

**Affiliations:** 1 Graduate School of Environmental & Symbiotic Sciences, Prefectural University of Kumamoto, 3-1-100 Tsukide, Higashi-ku, Kumamoto 862-8502, Japan; g2070005@pu-kumamoto.ac.jp (K.N.); g1975002@pu-kumamoto.ac.jp (E.T.); sshimoda@pu-kumamoto.ac.jp (S.S.); 2 Department of Nutrition and Metabolism, National Institute of Health and Nutrition, National Institutes of Biomedical Innovation, Health and Nutrition, 1-23-1 Toyama, Shinjuku-ku, Tokyo 162-8636, Japan; yhatamoto@nibiohn.go.jp; 3 Human-Centered Mobility Research Center, Information Technology and Human Factors, National Institute of Advanced Industrial Science and Technology, AIST Tsukuba Central 6, 1-1-1 Higashi, Tsukuba, Ibaraki 305-8566, Japan; takafumi.ando@aist.go.jp

**Keywords:** early time-restricted eating, early time-restricted feeding, late dinner, glucose fluctuation, substrate oxidation, appetite

## Abstract

**Aim:** To examine whether mild early time-restricted eating (eating dinner at 18:00 vs. at 21:00) improves 24-h blood glucose levels and postprandial lipid metabolism in healthy adults. **Methods:** Twelve participants (2 males and 10 females) were included in the study. In this 3-day (until the morning of day 3) randomized crossover study, two different conditions were tested: eating a late dinner (at 21:00) or an early dinner (at 18:00). During the experimental period, blood glucose levels were evaluated by each participant wearing a continuous blood glucose measuring device. Metabolic measurements were performed using the indirect calorimetry method on the morning of day 3. The study was conducted over three days; day 1 was excluded from the analysis to adjust for the effects of the previous day’s meal, and only data from the mornings of days 2 and 3 were used for the analysis. **Results:** Significant differences were observed in mean 24-h blood glucose levels on day 2 between the two groups (*p* = 0.034). There was a significant decrease in the postprandial respiratory quotient 30 min and 60 min after breakfast on day 3 in the early dinner group compared with the late dinner group (*p* < 0.05). **Conclusion:** Despite a difference of only 3 h, eating dinner early (at 18:00) has a positive effect on blood glucose level fluctuation and substrate oxidation compared with eating dinner late (at 21:00).

## 1. Introduction

Eating dinner late promotes metabolic dysfunctions including glucose metabolism in both rodents and humans [1,2,3]. In contrast, early time-restricted eating (ETRE), which requires eating the last meal of the day earlier, thereby lengthening the period of time that elapses before the next meal, appears to have a positive effect on blood glucose, insulin sensitivity, blood pressure, and triglycerides, especially in overweight or obese people [4,5,6,7]. The biological effects of ETRE, independent of energy restriction, have also been demonstrated in animal models [8,9,10,11].

The health benefits of ETRE or intermittent fasting seem to result from more than simply reduced free-radical production or weight loss, but the specific mechanism is not fully understood. The beneficial effects of intermittent fasting methods, including ETRE, appear to include metabolic switching induced by hunger and food consumption [12]. A recent systematic review also indicated that ETRE improves metabolic parameters such as blood glucose and blood pressure, and interestingly, decreases body fat mass independent of weight loss [13]. While the effects of ETRE are well-known, in the real world individuals do not appear to voluntarily restrict their eating habits to obtain these benefits.

According to the results from the NHANES study (2007–2010) conducted in the United States, 65% of individuals snack after dinner [14]. In addition, the average interval between the first and the last eating episode in a day is about 12 h in U.S. adults [14].

Management of fasting blood glucose levels is important for preventing diabetes and cardiovascular disease in healthy adults, and high fasting blood glucose levels, even those that fall within the normal range, predict the onset of diabetes and cardiovascular disease in the future [15,16,17]. Although fasting glucose levels is suitable for the assessment of chronic hyperglycemia, it does not accurately assess postprandial blood glucose, glycemic variability, and average glucose levels of a 24-h. Therefore, it is important to monitor how glycemic levels change over the course of a 24-h period. A previous crossover study showed that eating a late dinner resulted in higher glucose levels from after dinner until the next morning compared with consuming an earlier dinner, despite both meals having the same energy content [18], but the effect on blood glucose level fluctuations of over 24 h was not assessed. In addition, of the 23 studies included in a meta-analysis [13] performed to analyze the effects of time-restricted eating or intermittent fasting on metabolic disease, only one study evaluated 24-h glucose levels in a crossover design in healthy participants [7]. In a randomized crossover study performed by Jamshed et al., overweight adults who ate between 8 a.m. and 2 p.m. (ETRE) exhibited improved mean 24-h glucose levels and glycemic excursions compared with those who ate on the control schedule (between 8 a.m. and 8 p.m.) [7]. One drawback of this study, however, is that the window for food consumption was only 6 h, which is half of the 12-h food consumption windows observed in the real world. In addition, it is difficult for many individuals to eat their last meal of the day at 14:00, so it seems important to propose an ETRE schedule that is more achievable in real life. However, the evidence for mild ERTE intervention studies is clearly limited.

We showed in a previous study that increased lipid oxidation is a predictor of postprandial hyperglycemia [19]. Increased lipid oxidation by ERTE may affect glycemic regulation. It has also been shown in a study with a similar cross-over design that ETRE increases 24-h lipid oxidation (as assessed using a metabolic chamber), but does not increase energy expenditure [20]. Since lipid metabolism is promoted by lengthening the fasting time, this observation was attributed to the difference in the duration of the overnight fasting time (18 h in the ETRE group vs. 12 h in the control group). This large difference in fasting time may also affect the metabolic response after the next meal, typically breakfast; but interestingly, no significant difference in metabolic response was shown in this study [20]. Because of the 6-h difference in fasting time, differences in the amount of lipid oxidation that occurred before the next meal may have masked differences in the metabolic response after breakfast.

Thus, we hypothesized that mild ETRE would have a positive effect on blood glucose levels and postprandial lipid metabolism after the first meal of the day. Specifically, we investigated the effect of a difference of only 3 h between the final meal of the day (consumed at 18:00 or 21:00) on blood glucose levels and lipid metabolism.

## 2. Materials and Methods

### 2.1. Participants

The participants were non-smokers with no major diseases. In total, 14 participants (3 males and 11 females) over 20 years of age were included in the study. One of the participants dropped out in the middle of the study due to illness, and another dropped out due to a scheduling conflict. Participants were instructed not to drink alcohol the day before the blood glucose measurement. The sample size of 12 participants for this study was selected on the basis of a previous study [21] that investigated the effects of skipping breakfast but did not report the effects of eating a late dinner or record blood glucose level fluctuations. The effect size (Cohen’s d) was calculated to be 1.162 (alpha error 0.05, power 0.95). The required sample size was 12 participants, but we initially recruited 14 in anticipation of multiple dropouts.

### 2.2. Study Protocol

**Figure 1:**
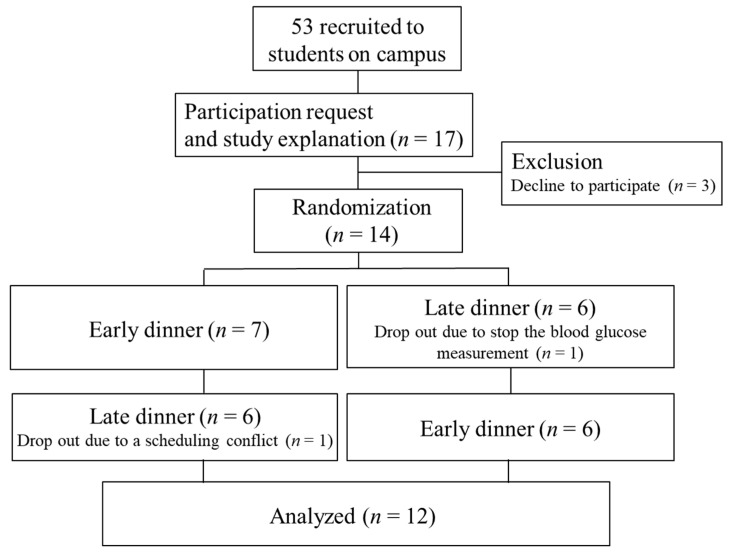
The flow diagram of the participant.

A participant flowchart is shown in Figure 1. Protocols for the meal timing interventions are shown in Supplemental Figure. This 3-day randomized crossover study compared two different conditions: eating a late dinner (at 21:00) vs. eating an early dinner (at 18:00). The participants were randomly allocated to two groups of seven participants each by E.Y. and K.N. using a random number generator. On the first day, the participants were given a prescribed diet and instructed to eat at the designated time in their normal environment. On the second and third days, they were provided with a prescribed diet and ate at a set time under laboratory conditions. Participants were allowed to drink as much water as they wished. The participants were asked to dedicate 15 min to each meal, and the order in which the different foods included in each prescribed meal were consumed was not specified. All participants were asked to refrain from exercising from the day before the 3-day experimental period began until the end of the 3-day period. The participants were instructed not to eat anything after 21:15 on the day before the experiment began, and to fast from the time they woke up until they visited the laboratory for testing at 7:30 on the second and third days of the experiment. Participants were allowed to consume only water after dinner. Participants were instructed to go to bed at 24:00 and wake up at 6:30, and record their sleep status. The wash-out period lasted at least 3 days (5 ± 2 days). The experiment was conducted between March 2019 and December 2020. The protocol was registered as a randomized controlled trial entitled “Effects of meal timing and content on glycemic fluctuations and metabolic responses” under the University Hospital Medical Information Network (UMIN) registration number UMIN000042542 and was approved by the ethical committee of the Prefectural University of Kumamoto (30–22). All participants signed an informed consent form describing the purpose, methods, and significance of the study. 

### 2.3. Experimental Schedule

Each participant wore a continuous blood glucose measuring device and a tri-axis accelerometer from the day before each 3-day experimental period began and throughout the 3-day period. The meals for day 1 of the experiment were provided the day before the experimental period began, and were consumed the following day at 8:00, 12:00, and 18:00 or 21:00. Participants recorded meal intake using a paper diary. On day 2, the participants visited the laboratory before breaking their fast to have their height and weight measured and collect their meals for that day. In addition, the participants’ subjective psychological status was evaluated by questionnaire (self-reported visual analog scores for various psychological factors) before breakfast, 30 min after breakfast, before lunch, 30 min after lunch, before dinner, 30 min after dinner, and at 23:00. On the third day of the experiment, the participants similarly visited the laboratory under fasting conditions, had their height and weight measured, and collected their meals for that day. In addition, the psychological questionnaire was filled out before breakfast and 30 min after breakfast. Before breakfast, after resting for 30 min the resting metabolic rate was measured for 10 min in the seated position. Participants were instructed to start eating breakfast at 8:00 and finish eating by 8:15. Then, the participants’ resting metabolism was evaluated by exhaled gas analysis (15 min, 30 min, 1 h, 2 h, 3 h, and 4 h after finishing breakfast). The participants remained at the laboratory control until all metabolic measurements were completed (until 12:30). The continuous blood glucose monitor and accelerometer were removed at the end of the 3-day experimental period. Day 1 was set as an intervention adjustment period to decrease any acute effects. Data from Day 2 and Day 3 of intervention were used in the analysis.

### 2.4. Anthropometric Measurements

Bodyweight and height were measured to the nearest 0.1 kg and 0.1 cm, respectively, in a fasting state the morning of day 2 and day 3 using a digital scale with a stadiometer (DC-250; Tanita Co., Ltd., Tokyo, Japan). BMI was calculated as kg/m^2^.

### 2.5. Subjective Appetite Assessment

Participants’ appetite (hunger, fullness, desire to eat, and capacity to eat) was evaluated subjectively using visual analogue scales [22]. These scales were completed on day 2 (before breakfast, 30 min after breakfast, before lunch, 30 min after lunch, before dinner, and 30 min after dinner) and day 3 (before breakfast and 30 min after breakfast). Hunger and fullness, desire to eat, and capacity to eat were rated on a 100-mm line preceded by the question.

### 2.6. Test Meal

Estimated energy requirements were calculated by multiplying the basal metabolic rate by physical activity levels. The estimated basal metabolic rate was calculated using the formula from Ganpule et al. [23]. Physical activity was assessed using the International Standardized Physical Activity Questionnaire prior to the survey [24]. Each meal contained between 1500 and 2500 kcal and was within 200 kcal of the estimated energy requirement. The same meal was provided for both the early dinner and the late dinner, and contained 15% protein, 25% fat, and 60% carbohydrate. Participants were instructed to each breakfast at 8:00, lunch at 12:00, a snack at 15:00, and dinner at 18:00 (early) or 21:00 (late). Participants were asked to consume each meal within 15 min, and were allowed to consume the various foods included in each meal in any order they wished.

### 2.7. Physical Activity

Steps were counted using a tri-axial accelerometer (HJA-750C Active style Pro; Omron Healthcare, Kyoto, Japan) [25]. Participants wore the accelerometer on their waists during the intervention period, except while sleeping or bathing. We only used step count data that were collected over a period of 600 min or more of wearing time.

### 2.8. Measurement of Energy Expenditure and Substrate Oxidation

Measurement of energy expenditure and substrate oxidation were performed using the indirect calorimetry method. Douglas bags were connected to the mixing chambers to measure VO_2_ and VCO_2_ gas concentrations using a mass spectrometer (ARCO 2000; Arco System, Chiba, Japan). The expired minute volume was measured using a two-barrel, drum-type respirometer (CR-20, Fukuda Inka, Tokyo, Japan), which was calibrated using an L syringe prior to testing. The volume of expired air was determined using a dry gas volume meter (Shinagawa, DC-5, Tokyo, Japan) and then converted to the volume under conditions of standard temperature, pressure, and dry gas (STPD). The gas exchange results were converted to energy expenditure using Weir’s equation [26]. Respiratory gas analysis was conducted using the mixing chamber method to evaluate the volume of expired air, and the O_2_ and CO_2_ fractions were analyzed by mass spectrometry (ARCO-1000; Arco Systems, Chiba, Japan). For each measurement on day 3 of the experimental period (before breakfast and 15 min, 30 min, 1 h, 2 h, 3 h, and 4 h after breakfast), the participant wore a gas mask, and the exhaled gas was collected in a Douglas bag for 5 min, twice. Participants were supervised to ensure that they remained at rest throughout the measurement period.

### 2.9. Continuous Blood Glucose Level Monitoring

During the experimental period (3 days), blood glucose levels were evaluated by each participant wearing a continuous blood glucose measuring device (FGM: Flash Glucose Monitoring, Abbott Japan Co., Ltd., Tokyo, Japan). The glucose level in the interstitial fluid was measured every minute, and the average value was recorded every 15 min. The data were analyzed using the FreeStyle Libre software application. Glycemic excursions were calculated using the Mean Amplitude of Glycemic Excursions (MAGE), which was tabulated using an Excel-enabled workbook called EasyGV [27], as described in a previous study [7]. The incremental area under the curve (iAUC) for postprandial blood glucose levels up to 3 h was evaluated using the trapezoidal rule. Average blood glucose levels after a meal were also evaluated up to 3 h.

### 2.10. Statistical Analysis

The data are shown as mean ± standard deviation. The study was conducted over three days; day 1 was excluded from the analysis to adjust for the effects of the previous day’s meal, so only data from the mornings of days 2 and 3 were used for the analysis. Two-factor repeated-measures analysis of variance (ANOVA) was used to determine the effects of the trial (early dinner vs. late dinner) and timing (before and 0–240 min after breakfast) on energy expenditure, respiratory quotient, and blood glucose levels. Paired t-tests were used to compare the two groups (early dinner vs. late dinner), with the statistical significance level set at less than 5%. All statistical analyses were performed using SPSS software (version 22.0 for Windows, IBM. Corp, Armonk, NY, USA).

## 3. Results

### 3.1. Body Composition

There was no significant difference in body weight and height evaluated under fasting conditions before breakfast on day 2 and day 3 between the two groups (*p* > 0.05). The average heights of the participants on day 2 and day 3 were 159.3 ± 8.5 and 159.5 ± 8.6 cm, respectively, for the early dinner group, and 159.4 ± 8.6 and 159.3 ± 8.7 cm, respectively, for the late dinner group. The average weights on day 2 and day 3 were 56.8 ± 13.2 and 57.1 ± 13.1 kg for the early dinner group and 57.0 ± 13.3 and 57.1 ± 13.2 kg for the late dinner group.

### 3.2. Sleep and Physical Activity

According to the sleep time of the self-recording of the participant, the participants went to bed on day 1 and day 2 at 23:59 ± 0:40 and 24:02 ± 0:41, respectively, in both the early and late dinner groups (*p* > 0.05). There was also no significant difference in wake-up time from day 1 to day 3 between the two groups (the early dinner group woke up at 6:38 ± 0:29 vs. 6:32 ± 0:21 for the late dinner group, *p* > 0.05). In addition, there was no significant difference in step counts throughout day 2 between the two groups (9147 ± 4866 steps/day in the early dinner group vs. 7771 ± 3134 steps/day in the later dinner group, *p* > 0.05).

### 3.3. Blood Glucose Level Mean Values and Fluctuations by Meal

**Figure 2:**
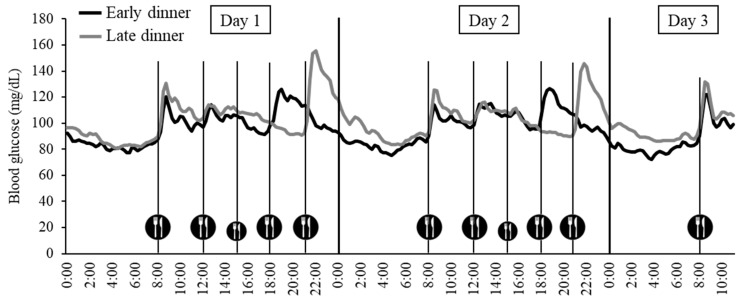
Blood glucose fluctuations from Day 1 to Day 3.

**Figure 3:**
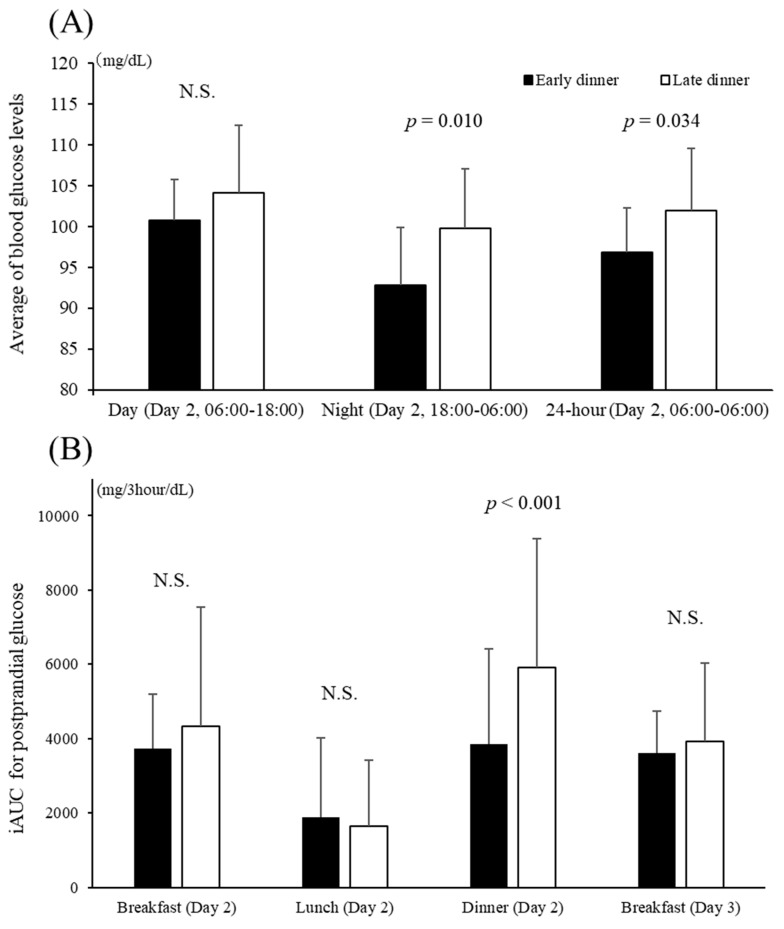
Average blood glucose levels (**A**) and iAUCs for postprandial glucose (**B**). Significant differences were observed in the mean blood glucose levels between the two groups throughout the day (*p* = 0.034) and for the 12-h period from night to early morning (from 18:00 on the second day to 6:00 on the third day) (*p* = 0.010). The iAUC for postprandial glucose was significantly higher for dinner on day 2 in the late dinner group compared with the early dinner group (*p* < 0.001). N.S.: not significant.

Figure 2 shows the blood glucose level fluctuations in both groups from day 1 to day 3, which were recorded every 15 min by the continuous blood glucose monitor. There was a more significant decrease in MAGE from day 1 to day 3 in the early dinner group than in the late dinner group (−8 ± 9 mg/dL, *p* = 0.027). Figure 3 shows the average blood glucose level and the iAUC up to 3 h postprandial glucose levels by a meal on day 2. Significant differences were observed in the mean blood glucose levels between the two groups throughout the day (*p* = 0.010) and during the 12-h period from night to early morning (18:00 on the second day to 6:00 on the third day) (*p* = 0.034). There was no significant difference in average blood glucose levels during the day (6:00 to 18:00) between the two groups. The iAUC for postprandial glucose levels was significantly higher for dinner on day 2 in the late dinner group compared with the early dinner group (*p* < 0.001), but there was no significant difference in the iAUC for postprandial glucose levels after breakfast or lunch (on days 2 and 3) between the two groups.

### 3.4. Changes in Energy Expenditure and Substrate Oxidation after Breakfast

**Figure 4:**
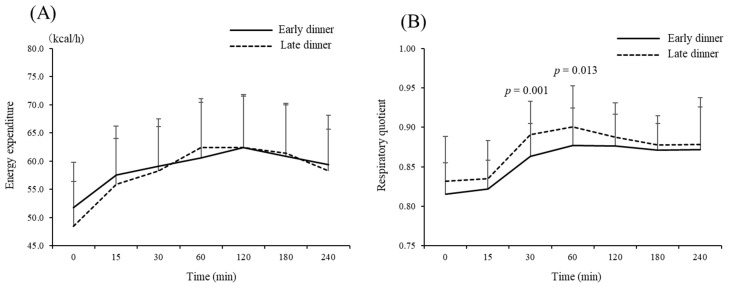
Effect of eating an early dinner on energy expenditure (**A**) and substrate oxidation (**B**) after breakfast the next day. An increase in energy expenditure after breakfast was observed in both groups (main effect, *p* < 0.05), but no significant difference was observed between the groups (*p* > 0.05). The postprandial respiratory quotient was significantly decreased 30 min and 60 min after breakfast in the early dinner group compared with the later dinner group (*p* < 0.05).

There was no significant difference in resting energy expenditure or respiratory quotient before breakfast between the two groups (Figure 4). An increase in energy expenditure after breakfast was observed in both groups (main effect, *p* < 0.05), but no significant difference was observed between the groups (*p* > 0.05). There was a significant decrease in the postprandial respiratory quotient 30 min and 60 min after breakfast in the early dinner group compared with the late dinner group (*p* < 0.05).

### 3.5. Subjective Appetite

**Figure 5:**
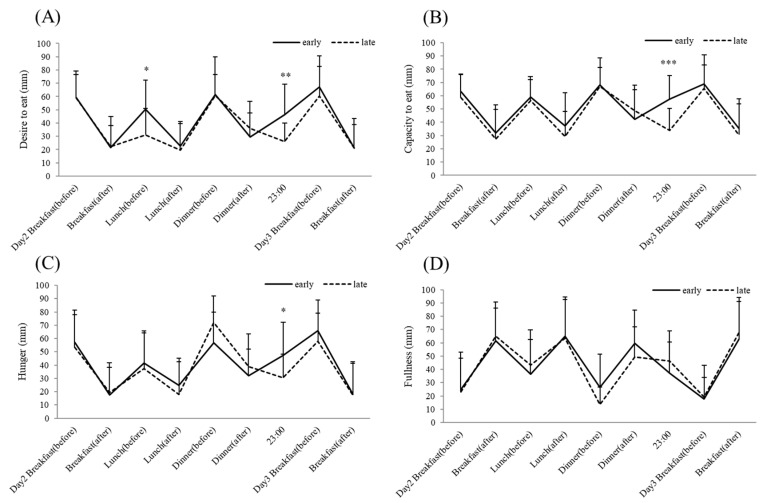
Eating dinner early modified temporal appetite patterns. (**A**) desire to eat, (**B**) capacity to eat, (**C**) hunger, (**D**) Fullness. * *p* < 0.05, ** *p* < 0.01, *** *p* < 0.001 (early dinner vs. late dinner), At timepoints without asterisks there was no significant difference between the two groups.

Assessment of subjective appetite was performed from before breakfast on day 2 to after breakfast on day 3 (Figure 5). There was no significant difference in subjective appetite between the groups for most of the experimental period, but the subjective scores for desire to eat, capacity to eat, and hunger recorded at 23:00 on day 2 were significantly higher in the early dinner group than in the late dinner group (*p* < 0.05). There was no significant difference in the subjective feeling of fullness between the groups at any time point measured.

## 4. Discussion

In this study, we hypothesized that (1) eating dinner early (at 18:00 instead of 21:00) would lower the blood glucose level, and (2) changing the timing of meals would affect substrate oxidation. We found that eating dinner early (at 18:00) lowered blood glucose levels after dinner and throughout the night compared with eating dinner late (at 21:00), even though the energy content was the same. Thus, this study shows for the first time that eating dinner only 3 h earlier (18:00 vs. 21:00) has a positive effect on 24-h glycemic control, as well as lipid metabolism after breakfast the following day, in healthy participants.

The mean 3-h blood glucose level after dinner was significantly higher in the late dinner group than in the early dinner group. Furthermore, in the late dinner group, the average blood glucose level from night until early morning (18:00 to 6:00 the next day) and the average blood glucose level over the entire second day (24 h) were higher than in the early dinner group. In contrast, in a study performed by Sutton et al. [5] in which men with prediabetes were assigned to ETRE (early time-restricted eating; 6-h eating period, with dinner before 15:00) or a control schedule (12-h eating period) for 5 weeks and then crossed over to the other schedule, showed that ETRE reduced fasting insulin and insulin resistance compared with the control schedule, but did not affect fasting blood glucose levels or glucose levels recorded during a 3-h oral glucose tolerance test. However, blood glucose levels were not measured by CGM for 24 h in this study; instead, they were only measured in the morning. Thus, it may not have been possible to detect effects that occurred from dinner to early morning, which showed the largest difference in our study. A recent systematic review and meta-analysis [4] found that studies including participants with metabolic abnormalities showed a significant change in fasting glucose concentrations (mean difference, −2.29; 95% CI, −4.29 to −0.19) depending on eating schedule. However, studies including only healthy participants did not show a significant change, the fasting glucose concentration tended to be lower (mean difference, −3.65; 95% CI, −7.65 to 0.35) [4]. Thus, when targeting participants with relatively small blood glucose fluctuations, such as healthy individuals, it may be important to monitor blood glucose fluctuations over 24 h in order to clarify the effects of ETRE.

In a cross-sectional study of patients with diabetes, late-night dinner consumption was associated with elevated hemoglobin A1c levels after adjusting for age, BMI, sex, duration of diabetes, smoking, and exercise [28]. Glycemic homeostasis also fluctuates throughout the day, and seems to be especially different in the early morning and at night. Decreased insulin sensitivity and glucose oxidation in the evening are caused by higher levels of postprandial free fatty acid levels in the evening compared to in the morning [29]. Previous studies have indicated that eating later in the day worsened postprandial hyperglycemic status, β-cell responsiveness to glucose, and hepatic insulin extraction in healthy adults compared with eating earlier in the day [30,31], and that appetite hormone and metabolite levels tended to be higher in participants who ate earlier in the day [30]. Previous studies have reported that insulin secretion was lower, and insulin resistance was improved, in the ETRE group compared with the control group [5,6]. The results from the current study seem to confirm these findings. However, blood insulin levels were not assessed in this study, so further investigation is required. In addition, this study focused on time and glucose metabolism, but daylight hours and other factors vary by season and country, and circadian rhythms of factors such as melatonin may differ accordingly. Because the circadian rhythm of melatonin secretion and that of insulin secretion are thought to be synchronized [32], and melatonin has also been reported to inhibit insulin secretion [33], future studies focusing on the circadian rhythm are also necessary.

Consistent with previous studies [20,32], this study found no significant difference between groups regarding the change in energy expenditure after breakfast. There was no difference in the respiratory quotient before breakfast on day 3 of the experimental period between the groups, but the respiratory quotient after breakfast was significantly lower in the early dinner group than in the late dinner group. The randomized controlled trial performed by Ravussin et al. [20] compared an ETRE schedule (eating between 8:00 and 20:00; 12-h daily eating period) with a control schedule (eating between 8:00 and 14:00; 6-h daily eating period) and found that prolonging the fasting time by restricting the daily eating period reduced the non-protein RQ for 24 h and increased the amount of lipid oxidation. These differences were driven by a lower non-protein RQ at nighttime, while sleeping, and while fasting in the morning. In contrast with the current study, there was no difference in non-protein RQ after breakfast, and there was also no significant difference during the daytime, after lunch and after dinner. However, we believe that this apparent difference in postprandial RQ results is clearly due to the difference in duration of the fasting period from dinner to the early morning of the next day.

Differences in the timing of the evaluation (19:00 vs. 23:00) and the participants (prediabetic adults vs. healthy adults) may account for apparent inconsistencies with some previous studies.

Sutton et al. found no significant difference in subjective measures of appetite such as desire to eat, capacity to eat, hunger, fullness, and stomach fullness in the morning between the ETRE and control groups. However, participants in the ETRE group reported significantly lower desire to eat and capacity to eat, as well as the significantly increased sensation of fullness in the evening in the preceding day (before dinner according to the control schedule (19:00)). Thus, despite the longer duration of the daily fasting period on the ETRE schedule, ETRE does not increase subjective hunger in the evening. Similar to previous studies, we found no significant difference in subjective appetite between the two groups in the morning (before and after breakfast) and in the afternoon. Surprisingly, even in the evening (before and after dinner), there was no significant difference in any of the subjective appetite items between groups. However, subjective appetite at 23:00 showed a significantly higher desire to eat, capacity to eat, and hunger in the early dinner compared with the late dinner. Previous studies, like ours, have reported that time-restricted feeding increases subjective appetite in the late evening [20,33]. Together, suppressing the urge to eat at night due to increased hugger may be an important factor in successful weight control.

Our study had some limitations. First, since the results from this study are transient, representing 3 days of intervention, long-term effects remain to be investigated. Second, due to the limited number of study participants, we were unable to draw any conclusions about sex-specific differences. Since there are sex differences in changes in lipid oxidation during fasting [34], and most of the participants included in previous studies of this topic were male [4], future studies should investigate the effects of sex differences. Third, although all of the participants were classified as healthy participants at the time of inclusion in the study, this was a subjective assessment that did not include an evaluation of the presence or absence of insulin resistance or habitual intake of dietary supplements. Additionally, we did not assess the effects of female participants’ menstrual cycles or measure serum insulin levels. Further research is needed to address these points in the future.

## 5. Conclusions

This study shows that, despite a difference of only 3 h, eating dinner early (at 18:00) resulted in higher average blood glucose levels the following day, a greater iAUC for blood glucose levels after dinner, and higher average blood glucose levels from evening to early morning (18:00 to 6:00) compared with eating dinner late (21:00). There was no difference in respiratory quotient before breakfast between the groups, but the early dinner group exhibited a lower respiratory quotient after breakfast and increased lipid oxidation compared with the late dinner group.

## Data Availability

The datasets generated during and/or analyzed during the current study are available from the corresponding author on reasonable request, pending ethics approval.

## Supplementary Materials

The following is available online at https://www.mdpi.com/article/10.3390/nu13072424/s1, Figure S1: Protocols for the meal timing interventions.
