# Supplementary figures and images for: Eating Dinner Early Improves 24-h Blood Glucose Levels and Boosts Lipid Metabolism after Breakfast the Next Day: A Randomized Cross-Over Trial
Source: Nutrients. 2021 Jul 15;13(7):2424. doi: 10.3390/nu13072424 (PMC8308587; doi:10.3390/nu13072424)

**Supplemental Figure S1.** Protocols for the meal timing interventions.

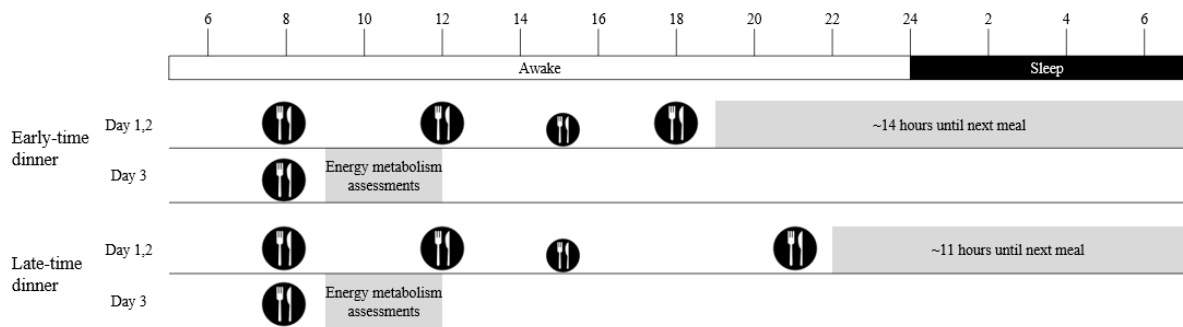

Supplement: Supplementary file 1 [file nutrients-13-02424-s001.zip › nutrients-1270582-supplementary.pdf]
